# Proteomics in Ataxia Telangiectasia Fibroblasts Revealed Disease Hallmarks Recovered by ATM Variants

**DOI:** 10.3390/ijms27188084

**Published:** 2026-09-11

**Authors:** Anastasia Ricci, Federica Biancucci, Gianluca Morganti, Muhammad Junaid Iqbal, Francesca Monittola, Mauro Magnani, Rita Crinelli, Michele Menotta

**Affiliations:** Department of Biomolecular Sciences, University of Urbino “Carlo Bo”, Campus Scientifico Enrico Mattei, Via Cà le Suore, 2, 61029 Urbino, PU, Italy; anastasia.ricci@uniurb.it (A.R.); federica.biancucci@uniurb.it (F.B.); g.morganti8@campus.uniurb.it (G.M.); m.iqbal@campus.uniurb.it (M.J.I.); francesca.monittola@uniurb.it (F.M.); mauro.magnani@uniurb.it (M.M.); rita.crinelli@uniurb.it (R.C.)

**Keywords:** ataxia telangiectasia, ATM variants, proteomics, phenotype rescue

## Abstract

Ataxia-telangiectasia (A-T) is a multisystemic genetic disorder caused by pathogenic mutations in the *ATM* gene, with effects extending beyond the canonical DNA damage response. We investigated whether two compact ATM transcript variants, ATM 4-53 (exons 4 and 53 joined) and ATM SINT (an in silico-designed ATM variant), could rescue disease associated with cellular alterations and inform construct selection for future delivery strategies. A-T fibroblasts transduced with either ATM variants or an empty vector were compared with wild-type fibroblasts using complementary label-free and tandem mass tag-based quantitative proteomics. Bioinformatic pathway and network analyses were integrated with biochemical and functional validation assays. A-T fibroblasts displayed coordinated alterations in proteostasis, proteasome function, RNA processing pathways, interferon-associated signaling, extracellular matrix organization, cell migration, and nanomechanical properties. Expression of both ATM variants partially or extensively reversed these abnormalities, restoring protein folding and proteasomal components, reducing protein aggregation, attenuating inflammatory signaling, and normalizing extracellular matrix-related phenotypes. ATM SINT generally produced the broader and more consistent rescue, particularly for proteostasis and RNA processing-associated networks. These findings show that compact ATM variants can recover multiple non canonical ATM dependent cellular functions in A-T fibroblasts and identify ATM SINT as the more effective construct in this model. Proteomic profiling combined with orthogonal functional validation may therefore support rational selection of ATM variants for subsequent preclinical delivery studies.

## 1. Introduction

Ataxia Telangiectasia, also known as Louis-Bar Syndrome (OMIM #208900), is a rare autosomal recessive multisystem condition caused by mutations in the ATM [1] gene. A-T primarily affects children and is characterized by progressive cerebellar ataxia, oculocutaneous telangiectasia, immunodeficiency, and predisposition to malignancies. A-T affects as many as 1 in 40,000 and 1 in 100,000 live births, with a median age of diagnosis of 6 years [2], and it has been recognized as a multifaceted disease with significant impacts on both neurological and systemic health. The condition arises from mutations in the ATM gene (ataxia telangiectasia mutated), which plays a crucial role in the cellular response to DNA damage, cell cycle control, and apoptosis [3,4,5].

The clinical manifestations of A-T are diverse and debilitating. Neurologically, patients exhibit gait disturbances, impaired coordination, and slurred speech, which progressively worsen over time. The hallmark telangiectasias, or dilated blood vessels, typically develop in the eyes and on the skin, further complicating diagnosis [6,7,8,9]. Immunologically, patients with A-T often experience recurrent respiratory infections resulting from both humoral and cellular immune deficiencies [10,11]. Furthermore, they face an elevated risk of malignancies, particularly lymphomas and leukemias, reflecting the genomic instability underlying the disease [12]. Despite advances in our understanding of the molecular mechanisms of A-T, therapeutic options remain limited, and clinical management is predominantly supportive. The administration of glucocorticoids via autologous erythrocytes has led to clinical improvement in patients with A-T [13,14], an approach that was recently reassessed in a phase 3 clinical trial [15]. Our team has investigated the molecular mechanisms underlying the action of dexamethasone in A-T [16,17,18,19,20,21,22,23], leading to the discovery of several ATM splice variants in patients’ blood [24]. Among these, ATM 3-52, ATM 4-53, mini-ATM, and the in silico-designed ATM SINT were biochemically characterized in an A-T cellular model [25].

Because the loss of ATM function is the primary cellular defect in A-T, several strategies have been explored to restore ATM expression in ATM deficient cells. Owing to the large size of the ATM cDNA (~9.2 kb), which exceeds the packaging capacity of most viral vectors, early approaches relied on HSV/AAV hybrid amplicon vectors to achieve targeted integration of the full-length human ATM cDNA [26]. Lentiviral vectors have since been used to reconstitute the A-T cellular phenotype in ATM deficient cells [27], and lentiviral ATM gene transfer has been specifically evaluated as a strategy for correcting ATM deficiency [28]. Alongside vector-based approaches, proof-of-concept studies are establishing hematopoietic stem cell-directed gene editing as a potential therapeutic avenue for A-T [29]. Collectively, these efforts demonstrate that reintroduction of ATM sequences is feasible, but they also highlight the need for compact, efficiently deliverable ATM constructs.

Omics-based approaches have already proven informative in A-T and related fields: proteomic and transcriptomic analyses of dexamethasone-treated A-T cells revealed extensive drug-induced molecular remodeling [19] and pathway-level analysis methods [30,31,32] allow the conversion of long lists of differentially expressed proteins into interpretable biological networks. Current research is focused on elucidating the overall phenotype reversion effects of the two best-characterized ATM variants, ATM 4-53 and ATM SINT, by a proteomic approach. Accordingly, the present study aimed to determine whether the expression of compact ATM variants can rescue the proteomic and functional alterations of A-T cells, and which of the two variants, ATM 4-53 or ATM SINT, displays the broader rescue capacity. The working hypothesis was that the two variants are not functionally equivalent, and that their differential rescue capacity reflects the presence of distinct internal structural domains. The omics analysis performed in this study made it possible to identify the most functional ATM variant for future applications involving gene delivery or gene therapy in an A-T animal model. Building on recent findings and ongoing research, these results highlight critical areas for future investigation and potential avenues for developing more effective interventions against this devastating disorder.

## 2. Results

### 2.1. Proteomic Outcome

**Figure 1:**
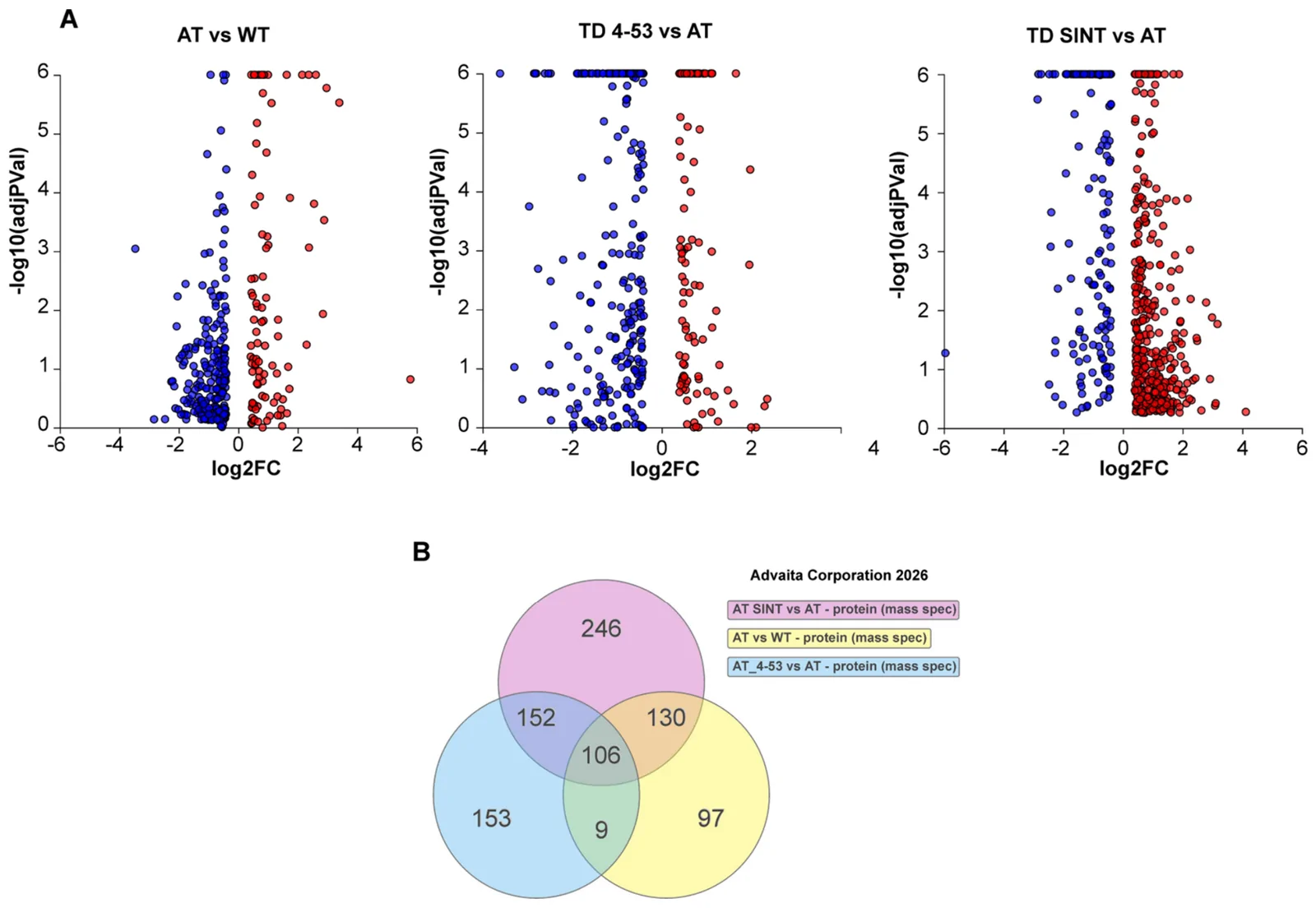
**Differential protein expression and overlap among experimental comparisons.** (**A**) Volcano plots showing differential protein expression in the three comparisons: A-T versus WT, ATM 4-53 versus A-T, and ATM SINT versus A-T. The x-axis represents log2 fold change (log2FC), whereas the y-axis represents −log10 of the adjusted *p*-value [−log10(adj-PVal)]. Upregulated proteins in red dot, downregulated proteins in blue. (**B**) Venn diagram showing the overlap and comparison-specific distribution of differentially expressed proteins identified in the three experimental comparisons. All differentially expressed protein features are reported in Supplementary File S1. A-T, ataxia-telangiectasia; WT, wild-type; ATM, ataxia-telangiectasia mutated; ATM 4-53, A-T cells expressing the ATM 4-53 variant; ATM SINT, A-T cells expressing the ATM SINT variant; log2FC, log2 fold change; adj-PVal, adjusted *p*-value. (also available in Figure S1).

**Figure 2:**
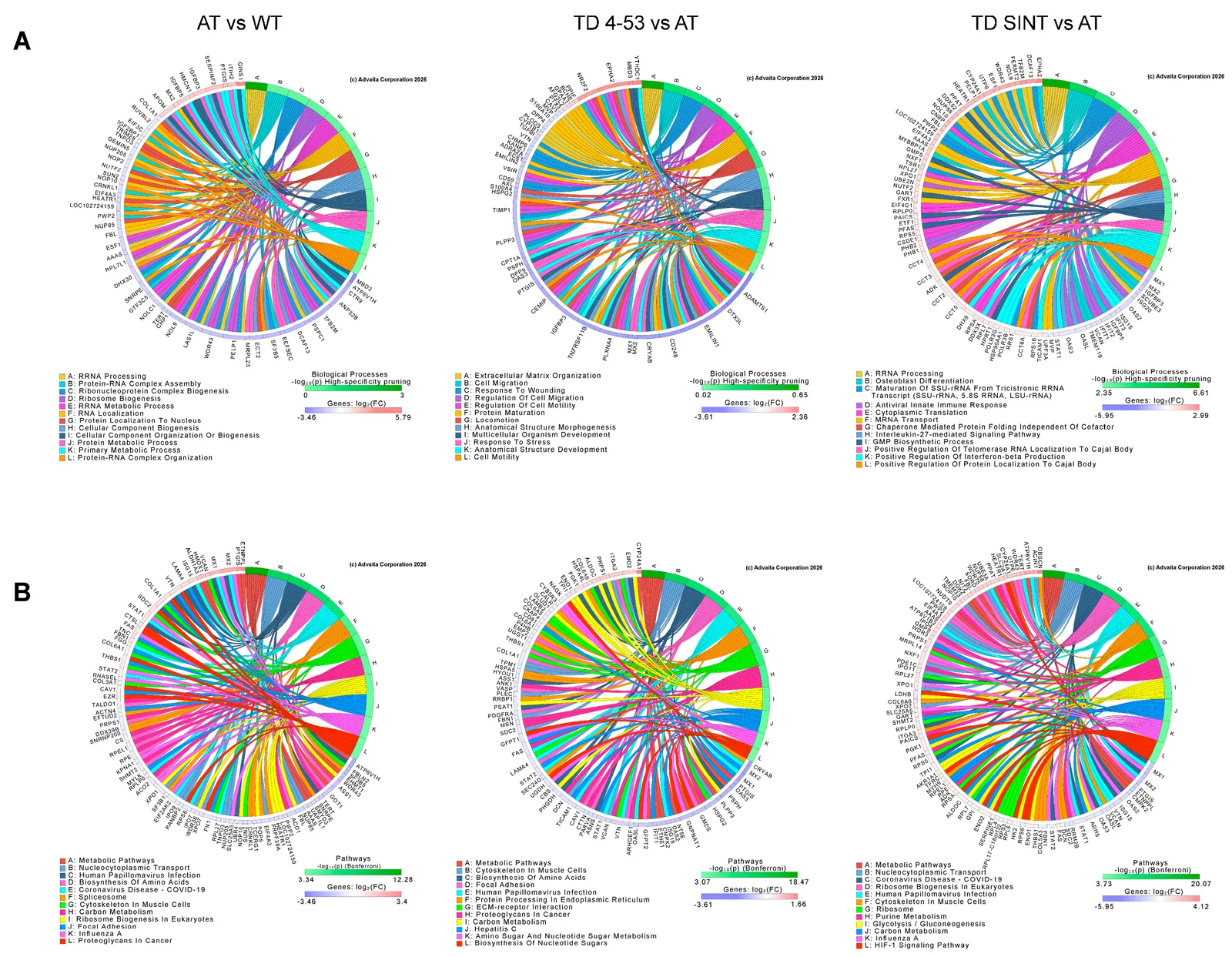
**Biological processes and pathways associated with differential protein expression in A-T fibroblasts and their modulation by ATM variants. **Chord diagrams showing the main biological processes (**A**) and pathways (**B**) identified by Advaita iPathwayGuide analysis in the three comparisons: A-T versus WT, ATM 4-53 versus A-T, and ATM SINT versus A-T. Biological processes were selected using the high-specificity pruning algorithm, whereas Bonferroni correction was applied to pathway analysis. A-T, ataxia-telangiectasia; WT, wild-type; ATM, ataxia-telangiectasia mutated; ATM 4-53, A-T cells expressing the ATM 4-53 variant; ATM SINT, A-T cells expressing the ATM SINT variant. (also available in Figure S1).

The overall effects of the ATM variants, ATM SINT and ATM 4-53, were evaluated by a proteomic approach. These two variants were previously tested in limited biological functions and resulted in outcomes that were more attractive to explore. WT, A-T UTD cells (transduced with empty vector), A-T SINT and A-T 4-53 transduced cells were employed in 2 independent bottom-up proteomic studies. Protein quantification was achieved by label-free and TMT (Tandem Mass Tag) isobaric labeling approaches. Through the label free based shotgun quantification, 3.430.627 MS2 spectra, 1.563.336 PSM (peptide spectrum match) 51.511 peptides groups and 5503 master proteins have been identified. The LFQ (Label-Free Quantification) normalization procedure among runs was successfully achieved. TMT-labeled fractions generated 2,522,378 MS2 spectra, 860.410 PSM, 99,888 peptide groups and 7848 master proteins. Isobaric labeling efficiency was over 93% for each sample. The comparison between A-T and WT led to the identification of 922 differentially expressed proteins; the comparison between TD 4-53, TD SINT and A-T brought out 1487 and 2033 expressed proteins, respectively. All the matching accession number protein of each condition (Supplementary File S1) alongside adj-P and log2FC were loaded in iPathwayGuide tool that scale down (isoforms reduction) the entries to 342 for WT vs. A-T, 420 for TD 4-53 vs. A-T and 634 for TD SINT vs. A-T (Figure 1A). The Venn diagram reported in Figure 1B shows the common and specific proteins in all tested comparisons. The first level analysis of proteomic data was obtained as a function of biological processes and pathways (High specificity pruning adj *p* < 0.05 and Bonferroni adj *p* < 0.05 correspondingly) as illustrated in Figure 2A and Figure 2B respectively. By examining the obtained KEGG (Kyoto Encyclopedia of Genes and Genomes) terms (using the Impact Analysis method [30,31,32]), some principal overall cellular occurrences were pointed out and subsequently evaluated by independent biochemical approaches.

### 2.2. Protein Folding and Proteasome Activity

**Figure 3:**
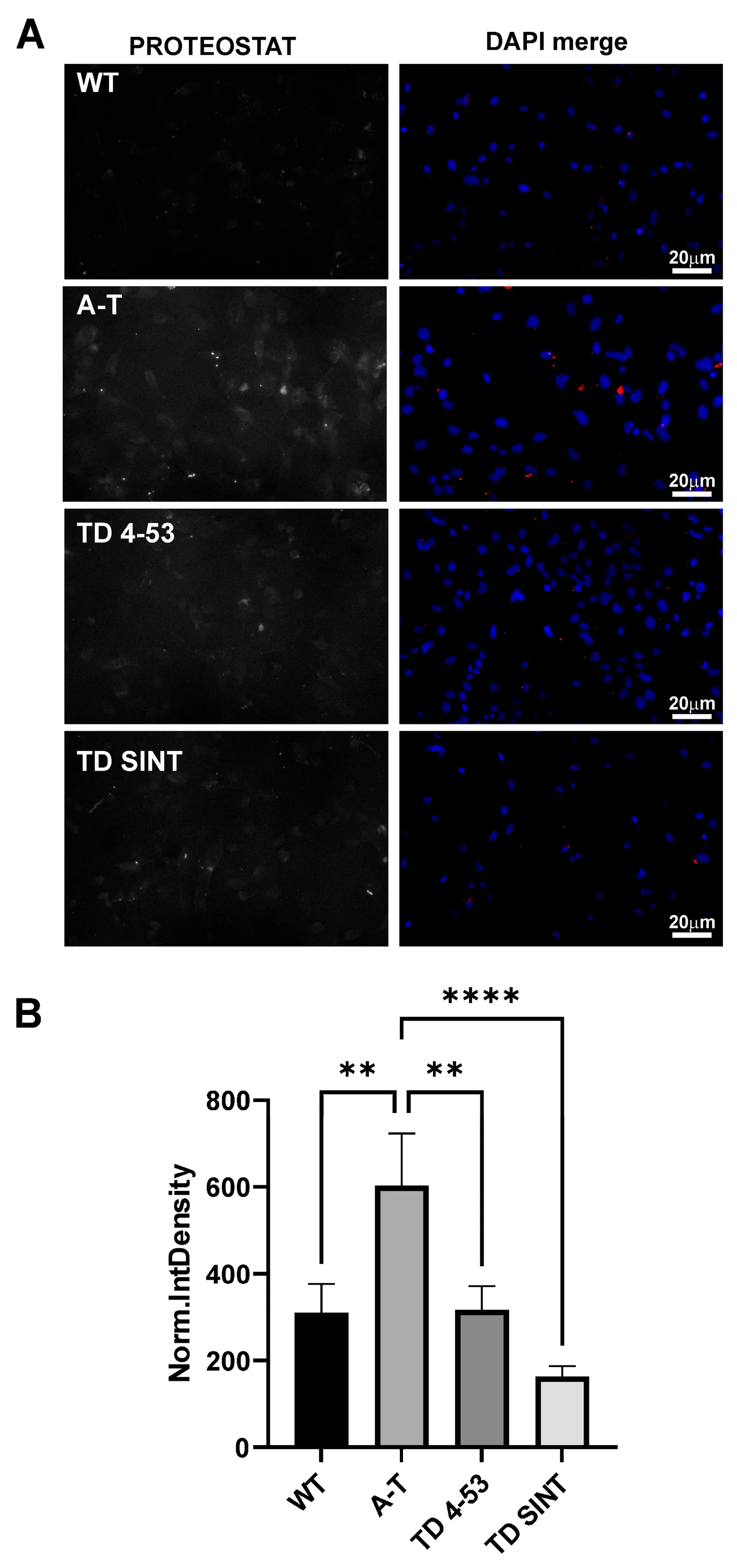
**ATM variants reduce protein aggregation in A-T fibroblasts.** The alteration of protein-folding homeostasis was functionally assessed by measuring whole-cell protein aggregates. (**A**) Representative images of PROTEOSTAT staining in WT, A-T, ATM 4-53, and ATM SINT cells. (**B**) Quantification of protein aggregates obtained from independent measurements. A-T cells showed a significantly higher amount of protein aggregates compared with WT cells (Fisher’s exact test, *p* = 0.0056), whereas expression of ATM 4-53 and ATM SINT significantly reduced protein aggregation toward WT levels (*p* = 0.004 and *p* = 0.0001, respectively). A-T, ataxia-telangiectasia; WT, wild-type; ATM, ataxia-telangiectasia mutated; ATM 4-53, A-T cells expressing the ATM 4-53 variant; ATM SINT, A-T cells expressing the ATM SINT variant; PROTEOSTAT, fluorescent dye-based assay for detection of aggregated proteins. ** *p* < 0.01, **** *p* < 0.0001.

This pathway was first evaluated since strictly related to previous observations about A-T autophagy impairments, and because this pathway is also included in the next described one. Several chaperone proteins and others involved in protein folding and stabilization (CCT5, CCT3, CCT4, CCT2, HSP90AA1, FKBP4, RANBP2, RUVBL2, DNAJB4, HYOU1, HSPD1, PPIF, QSOX2, KHSRP) were downregulated in A-T, while FKBP9, FKBP10, CRTAP, PRDX4 were upregulated. When ATM variants were expressed, the same proteins were modulated to WT levels (Supplemental Figure S2), especially in TD SINT cells. The alteration of the protein folding pathway was functionally verified by measuring the whole-cell protein aggregates as reported in Figure 3A,B. As suggested by proteomic analysis, the A-T cells showed higher amounts of misfolded proteins and ATM variants were both capable of reverting the observed phenotype.

Proteins strictly related to the protein folding pathway share the term “Cajal body,” determined as a pathway and biological process by iPathwayGuide. A subnetwork of this pathway is shown in Figure S3 (downregulated proteins in A-T) alongside the expression levels in the different conditions. TCP1 is a molecular chaperone that is a member of the chaperonin containing TCP1 complex (CCT), also known as the TCP1 ring complex (TRiC), located in the Cajal body organelle and responsible for spliceosome maturation [33].

TD SINT complementation successfully restores the missing proteins, while ATM 4-53 seemed to not affect this pathway. Alongside the Cajal body, the related “Spliceosome” pathway was downregulated (Bonferroni adj *p* = 0.015; Figure S4A–D). Consistently, we can suppose that the RNA processes were dysregulated and partially restored after ATM variant transduction.

**Figure 4:**
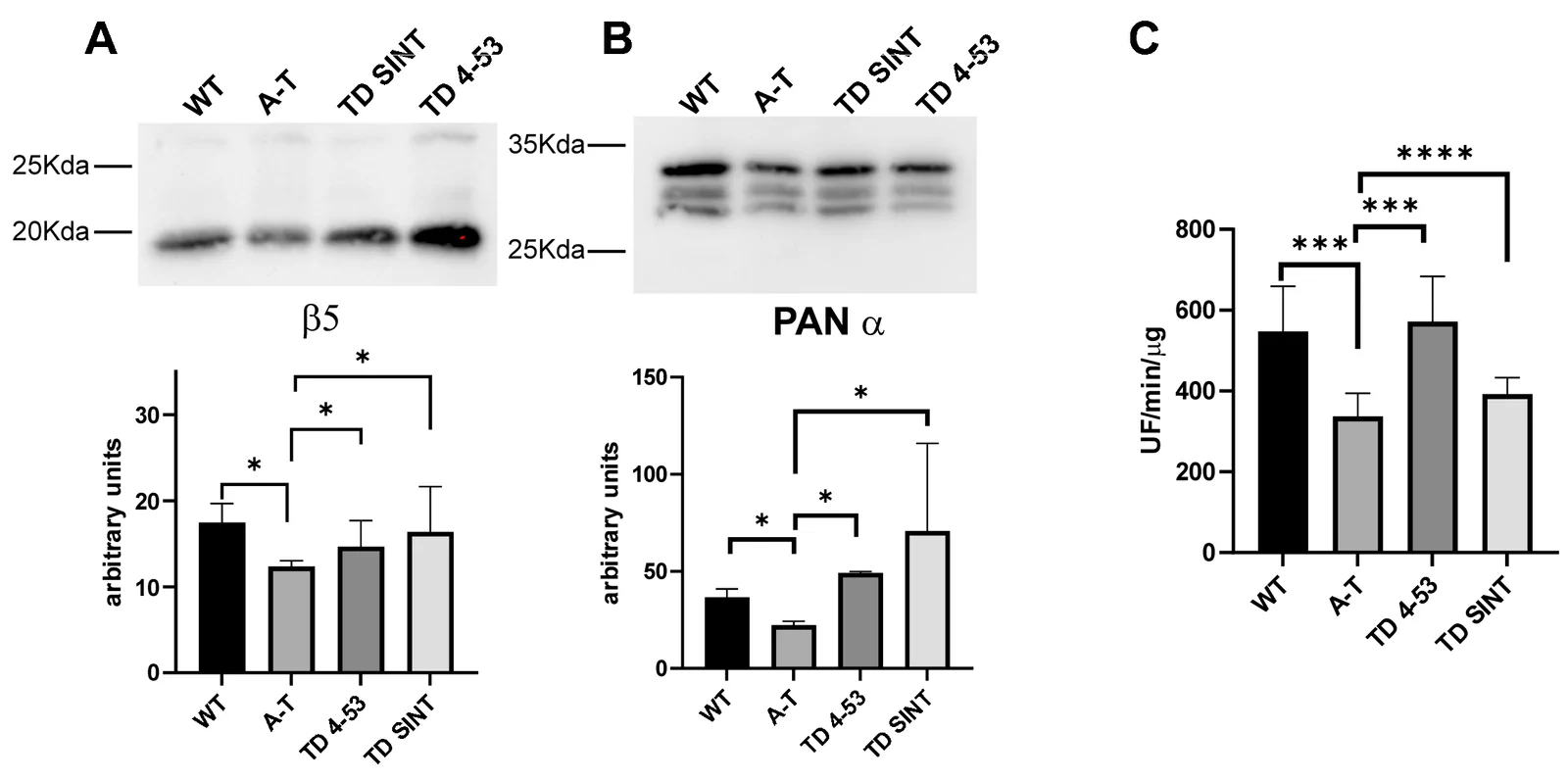
Proteasome subunits PSMB5 (**A**) and PAN-a (**B**) were also probed by Western blotting, confirming what was observed by omics data. PSMB5 was downregulated in A-T (Dunn’s test *p* = 0.023) and recovered in ATM 4-53 and ATM SINT transduced cells (*p* = 0.027 and *p* = 0.014, respectively). PAN−α subunit was down regulated in A-T (Dunn’s test *p* = 0.033) and recovered in ATM 4-53 and ATM SINT transduced cells (*p* = 0.027 and *p* = 0.027, respectively). In (**C**), the chymotrypsin activity of the isolated proteasome complex from all conditions. In A-T cells the activity was reduced than WT cell (Dunn’s test *p* = 0.0073) while the activity was restored to WT level in ATM 4-53 and ATM SINT transduced cells (*p* = 0.0001 and *p* = 0.0073 respectively). * *p* < 0.05, *** *p* < 0.001, **** *p *< 0.0001.

Since the accumulation of misfolded proteins can be caused by altered proteasomal activity, this process was also investigated. By pathway analysis, the term “Proteasome” emerged (Bonferroni adj *p* = 0.014). The regulatory subunits PSMB1, PSMB5, PSMB3, and PSME1 were downregulated, suggesting reduced proteasomal activity in A-T cells (Figure S5A,B). Since the number of proteins detected in this path, the PSMB5 subunit was also tested by Western blotting alongside the PAN-α ones (Figure 4A and Figure 4B, respectively). The tested subunits were deregulated in A-T cells (Dunn’s test *p* = 0.023) and restored by ATM variants (*p* = 0.027 and *p* = 0.027, respectively), thus confirming the proteomic outcome (Figure S5C). Also, the proteasome chymotrypsin activity was evaluated as reported in Figure 4C, and mostly the proteasome activity reflects the amount of its subunit quantity, contributing to the accumulation of misfolded proteins in A-T (Dunn’s test *p* = 0.0073). Once more, the ATM variants were able to restore the chymotrypsin activity (*p* = 0.0001 and *p* = 0.0073, respectively).

### 2.3. Response to Virus Infection and Interferon Alpha Terms

The chord diagrams in Figure 2A,B report several terms concerning the cellular response to virus infection and inflammation. Several protein belongings to these biological pathways are upregulated in A-T cells. These clusters are strictly related to the networks that refer to the cellular response to viral infection (Figure 2B). The activation of these pathways is very unusual, and in Figure S6 some of the involved proteins are reported.

To further investigate the activation of the suggested biochemical paths MX1 and phospho STAT3 (STAT3 not detected by proteomics) were further investigated by Western blot on independent samples and as reported in Figure S7, MX1 was upregulated in A-T cells and strongly decreased its amount in samples expressing the two ATM variants. STAT3 resulted in more phosphorylated A-T cells than WT and A-T expressing ATM variants, thus confirming the proteomic outcome.

### 2.4. Extracellular Matrix (ECM)

**Figure 5:**
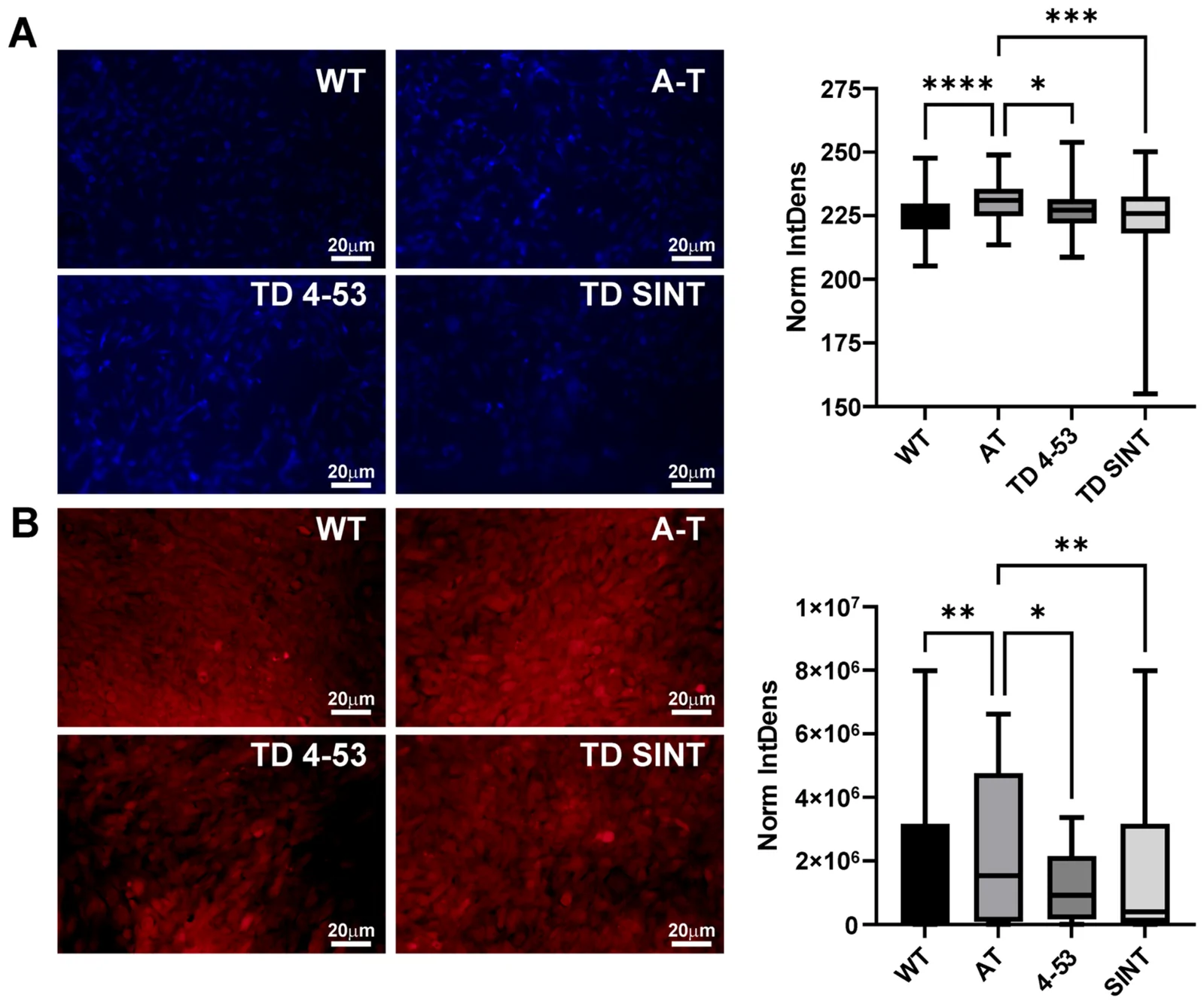
**ATM variants normalize altered extracellular protein deposition in A-T fibroblasts. **Extracellular protein deposition was evaluated using two independent fluorescence-based labeling approaches. (**A**) Representative images obtained by trichloroethanol-based Stain-Free labeling. Cells grown on coverslips were fixed and exposed to UV light to activate trichloroethanol-dependent protein fluorescence. (**B**) Representative images obtained using the No-Stain Protein Labeling Reagent after fixation of cells grown on coverslips. Images were acquired by fluorescence microscopy and analyzed by ImageJ. Quantification of extracellular protein-associated fluorescence is shown for each method. A-T cells displayed significantly increased extracellular protein staining compared with WT cells (Dunn’s test, *p* = 0.0001 and *p* = 0.0054 for Stain-Free and No-Stain labeling, respectively). This alteration was significantly reduced in ATM 4-53-expressing cells (*p* = 0.03 and *p* = 0.046, respectively) and in ATM SINT-expressing cells (*p* = 0.0004 and *p* = 0.005, respectively). Scale bars are shown in panels A and B. A-T, ataxia-telangiectasia; WT, wild-type; ATM, ataxia-telangiectasia mutated; ATM 4-53, A-T cells expressing the ATM 4-53 variant; ATM SINT, A-T cells expressing the ATM SINT variant. * *p* < 0.05, ** *p* < 0.01, *** *p* < 0.001, **** *p *< 0.0001.

From biological processes and pathways, it was possible to find several proteins involved in extracellular matrix dynamics, focal adhesion and collagen organization as deduced from the A-T cell sample. The involved proteins are reported in Figure S8, and the dysregulated amount in A-T cells was regulated by ATM variants. To further prove the proteomic outcome, the extracellular proteins were stained as reported in Figure 5A,B. The stain showed a different marked pattern in A-T cells compared with WT (*p* = 0.0001 for TAC assay, *p* = 0.0054 for no stain assay) whose amount was regularized in the ATM 4-53 cell line (*p* = 0.03 and *p* = 0.046, respectively) and in ATM SINT cells (*p* = 0.0004 and *p* = 0.005, respectively).

**Figure 6:**
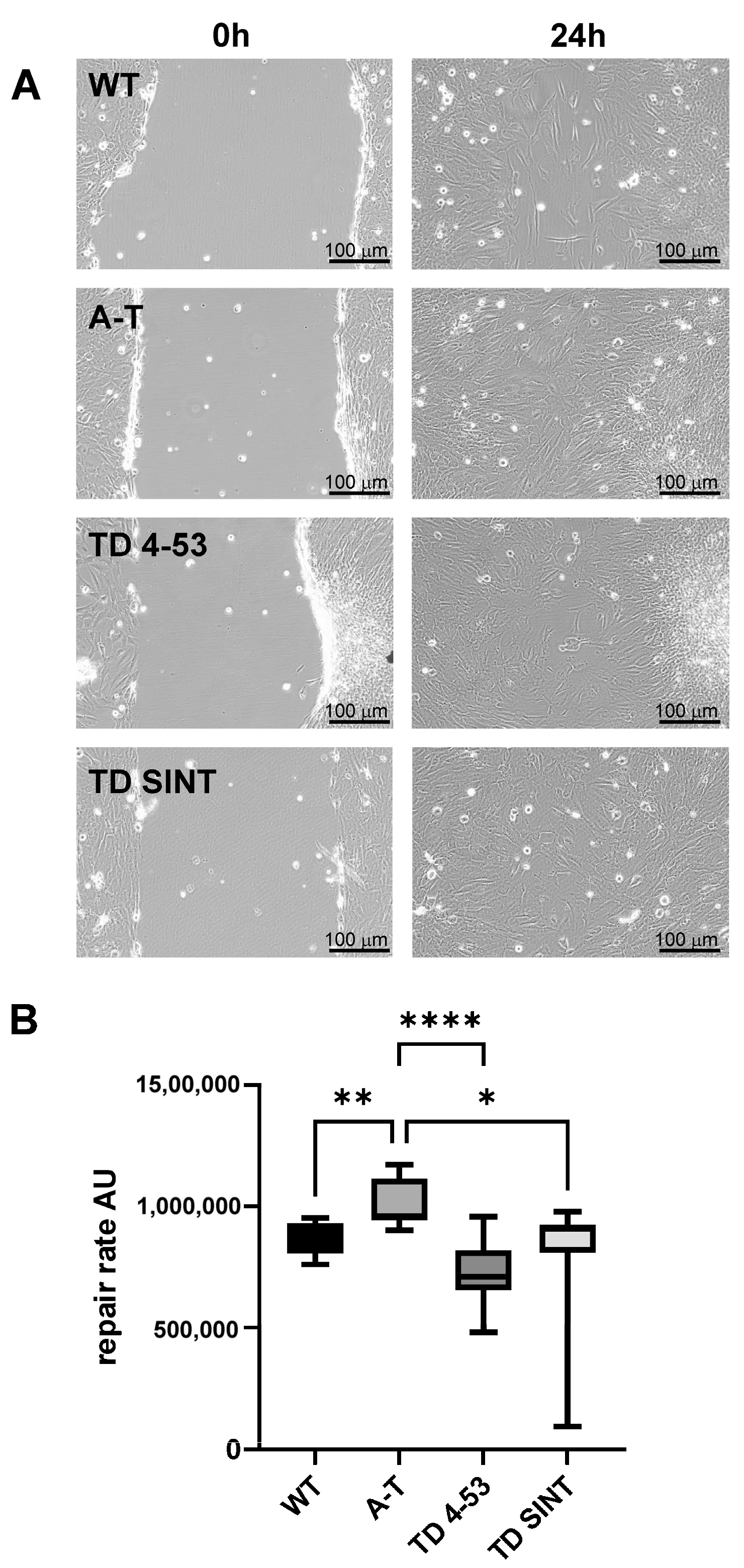
**ATM variants normalize the increased migratory behavior of A-T fibroblasts.** A scratch-wound assay was performed to evaluate cell migration in WT, A-T, ATM 4-53, and ATM SINT fibroblasts. Cells were grown to confluence and a linear scratch was generated across the cell monolayer using a sterile pipette tip. Detached cells were removed by washing, fresh medium was added, and images of the wound area were acquired at 0 h and at the indicated subsequent time points using an inverted microscope. (**A**) Representative images of the scratch area acquired at the indicated time points after wound generation. (**B**) Quantification of cell migration velocity. A-T cells displayed a significantly higher migration index compared with WT cells (Friedman test, *p* = 0.007). Expression of ATM SINT and ATM 4-53 significantly reduced this altered migratory phenotype toward WT levels (*p* = 0.02 and *p* = 0.0001, respectively). Scale bars are shown in panel (**A**). A-T, ataxia-telangiectasia; WT, wild-type; ATM, ataxia-telangiectasia mutated; ATM 4-53, A-T cells expressing the ATM 4-53 variant; ATM SINT, A-T cells expressing the ATM SINT variant. * *p* < 0.05, ** *p* < 0.01, **** *p *< 0.0001.

Since ECM remodeling can alter the growth of the cells, a scratch test was also performed in all the cell lines. In Figure 6A, a typical image of the experimental outcome is reported, and in Figure 6B, the cell migration velocity is reported. It is possible to appreciate that A-T cells have a greater migration index than WT (Friedman test *p* = 0.007) and that ATM SINT and ATM 4-53 are capable of modulating WT manner the growth (*p* = 0.02 and *p* = 0.0001 respectively).

AFM measurements performed in air with silicon nitride tips revealed a consistent reduction in pull-off forces in A-T fibroblasts compared with WT controls. Contextually, the stiffness of the cell surfaces was monitored, and it was possible to ascertain that the A-T cell surface was softer than that of WT cells. Concerning the last outcome, several proteins involved in submembrane cytoskeleton organization were downregulated in A-T (ACTN1, ACTN4, EZR). The two altered phenotypes were restored when the ATM variants were expressed in A-T cells (Figure S9).

## 3. Discussion

The present proteomic and functional analyses show that A-T fibroblasts have coordinated abnormalities extending beyond the canonical DNA damage response. These abnormalities involve proteostasis, RNA processing, interferon-related signaling, extracellular matrix organization, and cellular mechanical properties. Expression of the compact ATM variants ATM 4-53 and ATM SINT partially or substantially restored several of these alterations, with ATM SINT generally showing the stronger effect.

A major alteration involved protein quality control. Several components of the TRiC/CCT chaperonin complex and the proteasome were downregulated in A-T cells. These molecular changes were accompanied by increased protein aggregation and decreased chymotrypsin-like proteasomal activity, thereby providing functional support for the proteomic data. Expression of both ATM variants ameliorated these abnormalities, although ATM SINT yielded the most consistent recovery. These findings are in line with previous reports associating ATM dysfunction with impaired autophagy and disrupted cellular protein quality control [20,34] and with our earlier studies demonstrating that dexamethasone can partially restore autophagic and mitochondrial functions in A-T cells [19,25]. Although pathways such as autophagy and mTORC1 may contribute to the observed phenotype, they were not directly examined in the present study, and their specific involvement remains to be established experimentally.

The two ATM variants differed in their capacity to restore proteins associated with proteostasis. ATM SINT retains a larger internal segment of ATM than ATM 4-53 and consequently includes additional structural regions that may contribute to protein–protein interactions. Previous studies have reported interactions involving ATM, HSP90, and specific ATM structural domains [35,36]. These findings offer a possible basis for the distinct rescue profiles observed between the two variants. However, variant-specific interactions with HSP90 and the contribution of individual HEAT-repeat regions were not directly examined in the present study. Therefore, the structural features underlying the broader activity of ATM SINT remain to be elucidated.

RNA-associated pathways were likewise perturbed. Proteomic profiling revealed changes in proteins involved in Cajal body organization and spliceosome function in A-T cells, whereas expression of ATM SINT substantially shifted the abundance of several of these proteins toward WT levels. Previous studies have established functional links among ATM signaling, WRAP53β, Cajal bodies, and RNA processing pathways [37,38,39], thereby supporting the biological relevance of these findings. However, WRAP53β abundance, subcellular localization, phosphorylation status, and spliceosome activity were not directly assessed in the present study. Accordingly, our results indicate an association between ATM variant expression and the restoration of RNA-processing-related protein profiles but do not demonstrate a specific underlying molecular mechanism. The increased levels of PRPF38A, SF3B5, and YTHDC1 in ATM 4-53 expressing cells may reflect an adaptive or compensatory response; however, this interpretation requires further experimental validation.

A-T cells also exhibited a marked interferon- and antiviral response-associated signature. The proteomic findings were independently supported by increased MX1 expression and STAT3 phosphorylation, and both ATM variants attenuated these alterations. This observation is consistent with inflammatory signatures previously reported in A-T patient samples and experimental models [40,41]. Previous studies have implicated DNA damage associated with innate immune pathways, including cGAS-STING signaling, in ATM deficient conditions [42,43]. These mechanisms may contribute to the phenotype observed here; however, cGAS ubiquitination, RNF168 recruitment, and STING/IRF3 activation were not directly assessed. Accordingly, our data demonstrate that ATM variants attenuate the interferon-associated phenotype but do not establish the molecular pathway responsible for this effect.

Alterations in proteostasis and inflammatory signaling may also be interconnected, as previously described in other cellular stress conditions [44,45]. In our model, restoration of proteasome-associated functions and reduction in protein aggregation occurred together with attenuation of the interferon-associated signature. Although these parallel effects are compatible with functional crosstalk between the two processes, the present experiments do not establish a direct causal relationship.

A further major finding concerned ECM organization. A-T fibroblasts exhibited altered expression of several ECM-associated proteins, including collagen isoforms, lumican, fibronectin, laminin, vitronectin and tenascin-associated components, together with increased extracellular protein deposition. Despite this increase, A-T cells displayed a higher migration rate in the scratch assay, suggesting that ECM composition and organization, rather than its total amount alone, may influence cell behavior. Alterations in CXCL12 and other ECM-associated factors are consistent with this modified migratory phenotype [46,47,48,49]. Both ATM variants partially normalized ECM-associated protein profiles, extracellular protein staining, and cell migration, with ATM SINT again producing a generally stronger effect. Signaling pathways known to regulate ECM remodeling, including TGF-β, may contribute to these responses, although they were not directly investigated in the present study.

AFM analysis provided an additional functional perspective on the ECM and cytoskeletal findings. Compared with WT cells, A-T fibroblasts exhibited reduced pull-off forces and lower surface stiffness; both parameters shifted toward WT values following expression of the ATM variants. These changes coincided with alterations in ECM and cytoskeleton-linked proteins, including FN1, MYLK, ACTN1/4, and EZR. Reduced fibronectin abundance may contribute to the altered adhesive properties [50]. However, because the unfunctionalized Si_3_N_4_ probe measures nonspecific interactions, the specific contributions of individual ECM proteins cannot be resolved directly. The changes in surface stiffness are also consistent with the coordinated alterations in cytoskeletal proteins and with previous observations in A-T lymphoblastoid cells [17].

A limitation of the present study is that the experimental analyses were performed in an in vitro fibroblast model. Although this system allowed the molecular and functional effects of the ATM variants to be evaluated in an A-T cellular background, it does not fully recapitulate the cellular complexity and tissue-specific manifestations of the disease. Further validation in additional disease-relevant cellular models and, ultimately, in vivo models will therefore be required to establish the generalizability and therapeutic relevance of ATM 4-53 and ATM SINT.

Taken together, our results demonstrate that expression of compact ATM variants can recover multiple molecular and functional alterations of the A-T cellular phenotype. ATM SINT produced broader and more consistent rescue, particularly across proteostasis, RNA processing, and interferon-associated networks, whereas ATM 4-53 showed a more limited or pathway-dependent effect. Although the two variants differ in the internal ATM regions they retain, the present study does not establish which structural elements are responsible for their different activities. These findings therefore define the phenotypic consequences of ATM variant expression while providing a basis for future mechanistic studies. More broadly, they support the potential of engineered ATM variants as candidates for subsequent preclinical delivery approaches aimed at restoring both canonical and non-canonical ATM-dependent cellular functions.

## 4. Materials and Methods

### 4.1. Cells

Fibroblasts WT AG09429 (*ATM*+/+) and A-T GM00648 (*ATM*−/−) from the Coriell Institute (Camden, NJ, USA) were used as a cellular model. Cells were immortalized and transduced as previously described by Ricci et al. [25]. Briefly, A-T cells transduced with the variant ATM 4-53 was named TD 4-53, A-T cells transduced with ATM SINT was named TD SINT and A-T cells transduced with an empty vector were named UTD. Cells were grown in MEM (Sigma-Aldrich, M2279, St. Louis, MO, USA). The medium was supplemented with 10 mM Glucose, 2 mM l-glutamine, 100 U/mL penicillin, and 0.1 mg/mL streptomycin (Sigma-Aldrich G8644 and P0781) and 10% fetal bovine serum (Gibco, Thermo Fisher Scientific, Cat. No. 10270106, Waltham, MA, USA). All cells were grown at 37 °C with 5% CO_2_. The expression of the cloned ATM variants was verified in Ricci et al. [25].

### 4.2. Proteomic Analysis

In total, 2 × 10^5^ cultured cells of each distinct cell type were seeded into a 90 mm Petri dish. The cells grew until reaching 70–80% confluence. Upon achieving confluence samples were split in two batches separately processed by EasyPep MS Sample Kit (Thermo Scientific A40006, Waltham, MA, USA); one batch was dedicated for label free quantification (LFQ) bottom-up analysis while the other one was labeled with the TMT (Tandem Mass Tag) labeling option (16plex TMTpro, Thermo Fisher Scientific A44520), mixed in equimolar ratio, and fractionated by High pH Reversed Phase Peptide Fractionation Kit (Pierce 84868). Both batches were desalted and dried with a Speed vacuum centrifuge (Savant SPD121P). Prior to MS analysis, both batches (and fractions) were solubilized in 0.1% formic acid, and the peptide content was quantified through the quantitative colorimetric peptide assay (Thermo Fisher Scientific Pierce 23275).

One microgram of labeled fractions of each sample was injected into an UltiMate 3000 RSLC nano system coupled to the Exploris 240 mass spectrometer (Thermo Fisher Scientific) and resolved by Easy Spray Pepmap RSLC C18 (2 μm, 50 cm × 75 μm ES903) at a flow rate of 300 nL/min with a gradient of phase B (80% acetonitrile/0.1% formic acid, solvent A was 0.1% formic acid in water) from 2% to 40% in 100 min. Phase B was then changed up to 99% in 20 min, kept for 5 min, and then the column was re-equilibrated for 10 min.

Data was acquired in positive mode and in a data-dependent manner. For MS1, the *m*/*z* range was set to 350–1500 at 120,000 resolution (at *m*/*z* 200), AGC target 3 × 10^6^, and auto maximum injection time. MS2 was adopted when ion intensity was above 5 × 10^3^, with *m*/*z* range in auto mode, normalized HCD collision energy of 35%, AGC target 7.5 × 10^4^, and maximum injection time 40 ms. The resolution was set to 45,000 at *m*/*z* 200 and the internal calibrant was employed in run start mode. Fractions were analyzed in quadruplicate. For the LFQ batch samples (1.5 micrograms) were injected into the same system with a gradient of phase B from 2% to 50% in 200 min. Then (B) was changed up to 99% in 20 min, kept for 14 min, and then the column was re-equilibrated for 15 min. Data was acquired in positive mode and in a data-dependent manner. For MS1, the *m*/*z* range was set to 350–1500 at 120,000 resolution (at *m*/*z* 200), AGC target 3 × 10^6^, and auto maximum injection time. MS2 switched when ion intensity was above 5 × 10^3^, with *m*/*z* range in auto mode, normalized HCD energy 30%, AGC target 7.5 × 10^4^, and maximum injection time 40 ms. The resolution was set to 15,000 at *m*/*z* 200 and the internal calibrant was employed in run start mode. LFQ analysis was performed in septuplicate.

Raw data generated by Xcalibur 4.2 software (Thermo Fisher Scientific) were analyzed using Proteome Discoverer 2.5 (Thermo Fisher Scientific) using the sequential SEQUEST algorithm, counting TMT modification as fixed or variable, respectively. In both LFQ and isobaric analysis was considered, the fixed modification Cysteine carbamidomethylation and the variable serine, threonine, or tyrosine phosphorylation were considered, as well as variable oxidation (M) and deamidation (N, Q). The false discovery rate was evaluated by a target decoy strategy in a concatenated q-value manner. FDR (strict) was set as 0.01, while FDR (relaxed) was set as 0.05.

Differentially expressed proteins (|log2FC|and *p* < 0.05) were selected and individually evaluated. Fold changes, adjusted *p* and accession numbers were also used to perform pathway analysis using Advaita’s proprietary Impact Analysis method using iPathwayGuide software (v2023, Advaita Corporation, Ann Arbor, MI, USA).

### 4.3. Western Blot

Total proteins were extracted using the Protein Extraction Reagent Type 4 (Sigma-Aldrich). Cells were sonicated with 10 pulses of 15 s at 60 W by a Labsonic 1510 Sonicator (Braun, Melsungen, Germany) and clarified by centrifugation for 10 min at 14,000× *g*. Protein concentration was determined by the Bio-Rad Protein Assay, based on Bradford’s method. Ten micrograms of proteins were separated by SDS-PAGE (Novex, NP0321BOX, Thermo Fisher Scientific, Waltham, MA, USA) according to the Laemmli protocol [51] and then transferred to nitrocellulose (0.22 μm, Bio-Rad 1620112, Hercules, CA, USA) by wet transfer and Towbin blotting. Membranes were probed with the primary antibody Anti alfa proteasome 20S alfa1,2,3,5,6,7 subunits (MCP231 ENZO), anti MX1 (CST 37849) and anti pSTAT3 (CST 9145) diluted in 5% *w*/*v* nonfat dry milk or BSA if required, in TBST. The utilized secondary antibody was HRP anti-mouse or anti-rabbit (Bio-Rad 170-6515, 170-6516). Immunoreactive bands were recorded using enhanced chemiluminescence (Advansta K-12045-D50) acquisition by the ChemiDoc Touch Imaging System (Bio-Rad). The whole-lane normalization (WLN) strategy was adopted in Western blot analyses using a tri-halo compound for protein visualization [52,53,54]. Acquired images were analyzed by Image Lab software 5.2.1 (Bio-Rad).

### 4.4. Proteasome Activity Assay

Cells were lysed in native buffer consisting of 10 mM Tris-HCl pH 7.5, 10 mM NaCl, 10% (*v*/*v*) glycerol, completed at the time of use with 1 mM dithiotreithol, 2 mM ATP/MgCl_2_. The samples were subjected to vortexing on ice for 1 min, repeated 5 times and then centrifuged at 14,000× *g* for 10 min at 4 °C. One microgram of each sample was used to measure the proteasome activity using a fluorogenic substrate (Cayman Chemicals 10008119, Ann Arbor, MI, USA): s-LLVY-AMC (Suc-Leu-Leu-Val-Tyr-7-amido-4-methylcoumarin), specific for the chymotrypsin-like activity. The assay buffer consisted of 50 mM Hepes/KOH pH7.8, 10 mM KCl. The reaction was initiated by addition of fluorogenic peptide (100 μM). The release of AMC from peptidyl derivatives after hydrolysis was measured at 37 °C for 30 min in a fluorescence microplate reader (FLUOstar OPTIMA, BMG Labtech, Ortenberg, Germany) set to excitation/emission wavelengths of 355/460 nm. Proteasome activity was calculated from the slope after linear regression analysis of the values plotted as a function of time (R^2^ > 0.98).

### 4.5. Protein Aggregation Assay

Protein aggregates were evaluated using the Enzo PROTEOSTAT Protein (Farmingdale, NY, USA) aggregation assay (ENZ-51035-K100), in accordance with the procedure outlined in the assay manual. Protein aggregates were visualized by fluorescence microscope Olympus IX51 (Olympus, Tokyo, Japan) and images acquired by the ToupCam camera (ToupTek Europe, Landsberg am Lech, Germany) and elaborated and analyzed by ImageJ (version 1.54g) [55].

### 4.6. Extracellular Matrix Evaluation

Extracellular protein amounts were evaluated by two different applications: (1) the No-Stain Protein Labeling Reagent (Thermo Fisher Scientific A44717). Briefly, cells grown on a coverslip were fixed, after medium removal and PBS washing, with 4% formaldehyde for 10 min. Then 1 mL of No-Stain dye working solution was added and kept for 10 min. (2) by embedding the same prepared cells in a solution of trichloroethanol under 5 min of UV exposure. After washing, the coverslips were mounted and embedded with ProLong Antifade (Thermo Fisher Scientific P36930). Slides were observed and acquired images were analyzed as previously described. A scratch assay was performed to assess cell migration. Cells were seeded on 6-well plates and grown to confluence. A linear scratch was made across the cell monolayer using a sterile pipette tip. Detached cells were removed by washing with PBS, and fresh medium was added. Images of the scratch area were taken at 0 h and at subsequent time points using an inverted microscope. The closure of the wound area was measured to evaluate the rate of cell migration.

### 4.7. Atomic Force Microscopy Evaluation

The XE100 atomic force microscope (AFM; PARK Systems Inc., Santa Clara, CA, USA) was used in this study. The AFM was equipped with a 50-micrometer scanner controlled by the XEP 1.8.1 software. For cell imaging, the microscope was set in true non-contact mode, with the X-Y stage in a closed loop, while the Z scanner was set in the closed loop and high-voltage modes. The Z scanner resolution was set to 1.8 Å. The scan speed was set between 0.25 Hz and 1 Hz. The cantilevers used were NSC35 tips with a nominal spring constant of 16 N/m and a typical resonant frequency between 185 and 430 kHz. Force-distance (F/D) data were collected by randomly designing a matrix of 64 × 64 points over 2.5 micrometers of cell surfaces. The forward velocity was set at 20 nm/s, while the retrace velocity was set at 30 nm/s. F/D raw outcomes were processed by XEI software (PARK Systems Inc.).

## 5. Conclusions

This study highlights the multifaceted role of ATM beyond its canonical function in DNA damage repair, encompassing proteostasis, RNA processing, immune regulation, and extracellular matrix homeostasis. Proteomic and functional analyses demonstrate that the restoration of ATM activity through engineered transcript variants, particularly ATM SINT, can reestablish critical cellular networks that are severely disrupted in A-T. The differential rescue capacities of ATM SINT and ATM 4-53 underscore the importance of internal structural domains for ATM’s non-nuclear functions. These findings support the feasibility of using tailored ATM variants as therapeutic tools to address the complex molecular phenotype of Ataxia-Telangiectasia. This work lays the groundwork for future strategies aimed at targeted delivery of mini-ATM constructs or mRNA-based variants to restore specific cellular functions in A-T patients, offering a promising avenue toward personalized and pathway-specific intervention.

## Data Availability

The original contributions presented in this study are included in the article/Supplementary Material. Further inquiries can be directed to the corresponding author.

## Supplementary Materials

The supporting information can be downloaded at https://www.mdpi.com/article/10.3390/ijms27188084/s1.

## Abbreviations

The following abbreviations are used in this manuscript:

ACTN1/4	Alpha-Actinin 1/4
adj-P	Adjusted *p*-value
AFM	Atomic Force Microscopy
AGC	Automatic Gain Control
AMC	7-amino-4-methylcoumarin
A-T	Ataxia-Telangiectasia
ATM	Ataxia-Telangiectasia Mutated
BSA	Bovine Serum Albumin
CCT/TRiC	Chaperonin Containing TCP-1/TCP-1 Ring Complex
COL1A1, COL3A1	Collagen Type I/III Alpha 1 Chain
DTT	Dithiothreitol
EZR	Ezrin
FC	Fold Change
FDR	False Discovery Rate
FN1	Fibronectin 1
GO	Gene Ontology
HEAT	Huntingtin, Elongation factor 3, protein phosphatase 2A, TOR1 domain
HRMS	High-Resolution Mass Spectrometry
HSP90	Heat Shock Protein 90
iPathwayGuide	Integrated Pathway Analysis Tool
KEGG	Kyoto Encyclopedia of Genes and Genomes
LFQ	Label-Free Quantification
LLVY-AMC	Suc-Leu-Leu-Val-Tyr-AMC (fluorogenic proteasome substrate)
MEM	Minimum Essential Medium
MS	Mass Spectrometry
MS2	Tandem Mass Spectrometry (second stage)
MX1	Myxovirus resistance protein 1
OMIM	Online Mendelian Inheritance in Man
PSM	Peptide Spectrum Match
PSMB1/3/5, PSME1	Proteasome subunits
pSTAT3	Phosphorylated STAT3
SDS-PAGE	Sodium Dodecyl Sulfate-Polyacrylamide Gel Electrophoresis
STAT3	Signal Transducer and Activator of Transcription 3
TBST	Tris-Buffered Saline with Tween 20
TCAB1	Telomerase Cajal body protein 1
TD 4-53	Transduced A-T cells expressing ATM 4-53
TD SINT	Transduced A-T cells expressing ATM SINT
TMT	Tandem Mass Tag
UTD	UnTransduced (A-T cells with empty vector)
WB	Western Blot
WLN	Whole Lane Normalization
WRAP53β	WD Repeat-Containing, Antisense to TP53 Beta
WT	Wild-Type
