# Supplementary material for: Proteomics in Ataxia Telangiectasia Fibroblasts Revealed Disease Hallmarks Recovered by ATM Variants
Source: Int J Mol Sci. 2026 Sep 11;27(18):8084. doi: 10.3390/ijms27188084 (PMC13607285; doi:10.3390/ijms27188084)
Supplement: Supplementary file 1 [file ijms-27-08084-s001.zip › caption.pdf]

International Journal of *Molecular Sciences*

MDPI

Supplementary File S1. Excel file contains all proteins differentially expressed in the A-T vs WT, ATM 4-53 vs A-T and ATM SINT vs A-T comparisons.

Figure S1. Enlarged high-resolution versions of the panels presented in Figures 1 and 2.

Figure S2. Expression level of proteins involved in the Protein folding term. The dysregulated proteins in A-T are remodulated by the ATM variants.

Figure S3. Subnetwork of proteins involved in Cajal body. Proteins are downregulated in A-T and up-regulated when ATM SINT is present. ATM 4-53 seems to not be involved in the restoration procedure, or its effects were slight and rejected by proteomics statistics.

Figure S4. In (A) spliceosome KEGG pathway. In (B) the A-T sample downregulated proteins and in (C) the functional subnetwork. In (D) the modulation of the patterned proteins by the two ATM variants that modulate this path, especially ATM SINT.

Figure S5. In (A) KEGG proteasome highlighted components. In (B) the expression pattern of the show up proteins in A-T sample. In (C) the modulation of proteasome by the two ATM variants. The activity of proteasome in tested samples is reported in Fig. 4C.

Figure S6. Proteins expression amount involved in "response to virus infection and interferon-alpha" terms. Altered expression pattern in A-T is modulated by the ATM variants.

Figure S7. Western blot of MX1 and pSTAT3, performed to confirm the KEGG activated path "response to virus infection and interferon-alpha" in A-T cells and to verify the ATM variants achievement in transduced cells.

Figure S8. Found-out ECM altered proteins in A-T samples and their modulation by ATM variants.

Figure S9. AFM F/D pull-off and stiffness data. In A-T the cell surfaces are quite different in terms of nanomechanical properties, and the ATM variants are capable of restoring WT similar properties.

https://doi.org/10.3390/ijms27188084
